# More than Moths: Flower Visitors of a Night-Blooming Plant in South Florida Pine Rocklands, USA

**DOI:** 10.3390/plants11202799

**Published:** 2022-10-21

**Authors:** María Cleopatra Pimienta, Suzanne Koptur

**Affiliations:** Department of Biological Sciences, Florida International University, 11200 S.W. 8th Street, Miami, FL 33199, USA

**Keywords:** butterflies, floral resources, *Guettarda scabra*, hawkmoths, insects, nectar robbing, pine rockland, pollination

## Abstract

Plants whose flowers open at night but remain open during the day also attract diurnal flower visitors, potentially boosting their pollination rates and providing resources that can support diverse arthropod communities. The rough-leaf velvetseed, *Guettarda scabra* (Rubiaceae), is an evergreen shrub that thrives only in the imperiled pine rockland habitat in south Florida. Its white, tubular, and fragrant flowers open during late afternoon, exhibiting traits strongly associated with the attraction of nocturnal hawkmoths (Sphingidae). Flowers of *G. scabra* remain open until the following morning, becoming available to a wider array of visitors, bringing into question the expectation that sphingophilous flowers are visited mainly by hawkmoths. To evaluate whether the flowers of *G. scabra* are mainly visited by nocturnal hawkmoths and understand the role of this plant in the pine rockland habitat, we characterized the arthropod fauna associated with its flowers during the morning, evening, and at night. We found that most flower visitors were diurnal insects of the orders Hymenoptera and Lepidoptera, although we observed other arthropod groups too. Visitation at night was dominated by two species of hawkmoths. Nectar was the main resource used by the arthropod community during this study. Legitimate visitation and nectar-robbing were the behaviors most frequently observed among the flower visitors. Our results suggest that flowers of the night-blooming *G. scabra* constitute an important food source for both diurnal and nocturnal arthropod fauna in the fire-dependent pine rocklands of southern Florida. Our study provides novel data to support efforts to conserve and protect pine rocklands and the plants and animals that inhabit them.

## 1. Introduction

Although floral morphology often suggests coevolution with determined pollen vectors, flowers usually attract other visitors too [1,2,3]. The availability of these visitors and the reproductive success of the plant are affected by the time at which flowers open and for how long they remain available for visits [4]. As such, night-blooming plants whose flowers remain open during the day are likely to receive diurnal visitations, boosting their pollination opportunities.

**Figure 1:**
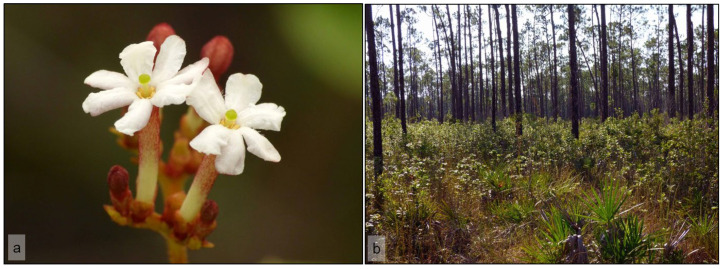
(**a**) Recently opened flowers of *Guettarda scabra*, during late afternoon. Some individuals, such as the one in this picture, have a long pistil that raises the stigma above the deep corolla tube. Exudates from the stigma were occasionally consumed by visitors such as flies, beetles, and possibly spiders during this study; (**b**) general view of pine rockland habitat at Long Pine Key, Everglades National Park in south Florida, USA. *Guettarda scabra* plants are abundant in patches scattered among *Pinus elliottii* trees.

The rough-leaf velvetseed (Figure 1a), *Guettarda scabra* (L.) Vent. (Rubiaceae), is a tropical evergreen shrub native to the Caribbean, ranging from the northern parts of Colombia and Venezuela to the southern portion of Florida (USA) [5,6,7,8]. In south Florida, *G*. *scabra* grows only in the last remnants of pine rockland (Figure 1b) and hardwood hammock habitats on the peninsular mainland, where it is abundant [9,10]. Pineland *G.scabra* plants are short in stature and allocate much more energy to flowering and fruiting than do the tall individuals persisting in hardwood hammocks [6]. 

*Guettarda scabra* flowers exhibit a set of traits associated with the attraction of nocturnal lepidopterans, particularly hawkmoths (Sphingidae). Sphingophilous flowers are pale, with long-tubed corollas, and emit a strong sweet scent [11]. Anthesis in *G. scabra* happens during late afternoon [12], which led to the assumption that they were exclusively for night-time visitors [13], particularly hawkmoths [10]. Recent observations have shown that these flowers remain open through the following morning and are visited by butterflies [14], suggesting that they can be attractive to other visitors too, providing resources to a larger arthropod community. Despite its local abundance, and its presence in the disappearing pine rocklands, the structure of the community of flower visitors associated with *G. scabra* has not been studied in detail, even though *G. scabra* thrives in an imperiled habitat and allegedly depends upon pollinators whose populations may be declining [15]. 

To test the hypothesis that flowers of this species are mainly visited by nocturnal Lepidoptera, we observed flowering plants during day and night. Besides nocturnal lepidopterans, we expected to find many other visitors to the flowers, not only at night, but evening and morning, during times the flowers are open, but hawkmoths are not present. We thoroughly characterize the local arthropod fauna associated with flowers of *G*. *scabra*, their behavior, and floral resources they use. We offer insights into the role played by this native plant species in its rockland habitat and identify many *G*. *scabra* potential pollinators, providing the basis for a deeper understanding of its pollination biology and its role in supporting the arthropod community of this imperiled ecosystem. By learning more about the relationships *G. scabra* has with pine rockland fauna, we test the traditional view of pollination syndromes and also elucidate the multitude of interactions a single plant species may have. In this approach, our study may reach beyond its local rare habitat and be relevant to other plant species worldwide. 

## 2. Results

**Figure 2:**
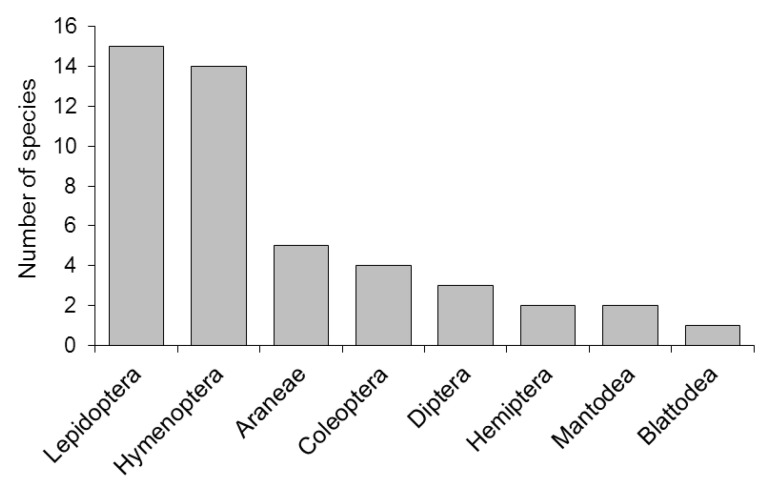
Arthropod orders observed on flowers of *Guettarda scabra*, sorted according to the number of species found. The large numbers of lepidopterans and hymenopterans are mostly related to diurnal activity in these two groups.

Flowers of *G. scabra* were visited by 46 species of arthropods, belonging to 8 orders and 20 families (Table 1). Most visitors were insects from the orders Lepidoptera and Hymenoptera (27 species total, vs. 17 other species; Fisher’s exact test *p* < 0.01), making up 63% of all species recorded. The proportions of visitors in these two orders did not differ significantly (Fisher’s exact test *p* > 0.05). The remaining were arachnids of the order Araneae, or insects belonging to the orders Coleoptera, Diptera, Hemiptera, Mantodea, and Blattodea (Figure 2).

### 2.1. Occurrence

**Figure 3:**
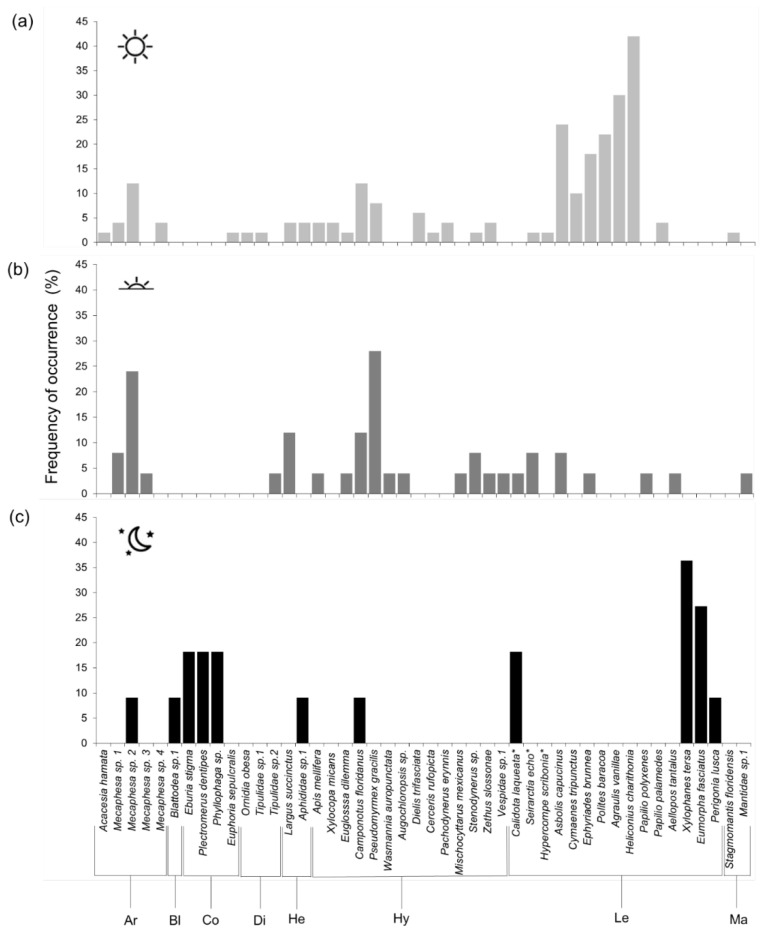
Frequency of occurrence of each visitor species on flowers of *Guettarda scabra*, relative to the total number of observation periods carried on during the (**a**) morning (*N* = 50), (**b**) evening (*N* = 25), or (**c**) night (*N* = 11). Ar: Araneae; Bl: Blattodea; Co: Coleoptera; Di: Diptera; He: Hemiptera; Hy: Hymenoptera; Le: Lepidoptera; Ma: Mantodea. Asterisks (*) refer to caterpillars.

Most species were found only in one of the study sites: 61% of the total number of species observed at Larry and Penny Thompson Park were unique to that site; 24% of species observed at Everglades National Park were observed only there. Only a small fraction of the total species observed (15%) was common to both sites (Table 1). The proportion of unique species observed at each site (80% at LPT, 61% at ENP) did not, however, differ significantly with Fisher’s Exact Test. Most arthropods registered (76%) were seen exclusively during daytime (especially the morning hours), substantially more (Fisher’s Exact test *p* < 0.01) than those observed to visit only at night (15.2%). An even smaller proportion (8.7%) of the species visited flowers both day and night (Figure 3). Overall, visitors were observed 3x more frequently in the daytime observations than in the evening observation periods, and 6x more frequently than during the night. Lepidoptera were the order most commonly observed during the morning and night; Hymenoptera most commonly in the morning and more than twice as often as Lepidoptera in the afternoon. Araneae, Diptera, and Hemiptera much more common in morning and evening; Coleoptera most often observed at night.

Flowers of *G. scabra* were visited in the morning mainly by two butterfly species, *Heliconius charithonia* and *Agraulis vanillae*, and three species of skippers, *Asbolis capucinus*, *Polites baracoa,* and *Ephyriades brunnea* (Figure 3). Evening visits were dominated by *Pseudomyrmex gracilis* ants and crab spiders of the genus *Mecaphesa*; while at night the hawkmoths *Xylophanes tersa* and *Eumorpha fasciatus* showed the highest occurrence (Figure 3).

### 2.2. Visitor Behavior

**Figure 4:**
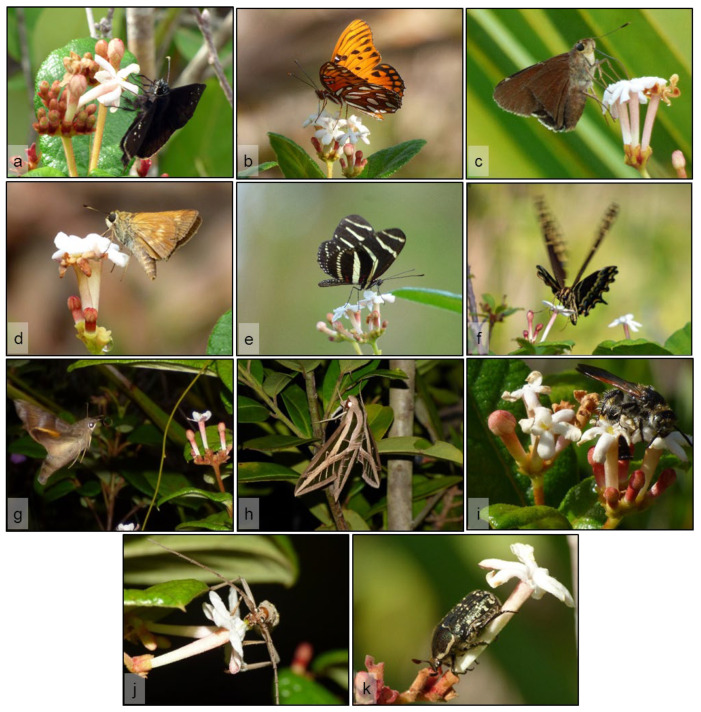
Overview of some species of flower visitors performing legitimate visitation and behaving as potential pollinators of *Guettarda scabra* at two pine rockland sites (Larry and Penny Thompson Memorial Park, LPT; and Long Pine Key, Everglades National Park, ENP) in south Florida, USA. Some lepidopterans such as (**a**) *Ephyriades brunnea*, (**b**) *Agraulis vanillae*, and (**c**) *Asbolis capucinus* were observed in both study sites, while (**d**) *Polites baracoa* and (**e**) *Heliconius charithonia* were seen only in LPT. Other visitors were only seen in ENP, such as (**f**) *Papilio palamedes* that feeds on nectar during daytime, and the nocturnal hawkmoths (**g**) *Perigonia lusca* and (**h**) *Eumorpha fasciatus*, represented here by an individual resting after a feeding bout. Besides lepidopterans, (**i**) the wasp *Dielis trifasciata* is seen here coming in close contact with the exposed stigma of a flower as it feeds on pollen during the morning. Beetles such as (**j**) *Eburia stigma* and (**k**) *Euphoria sepulcralis* visited flowers to feed on pollen and stigma exudates.

**Figure 5:**
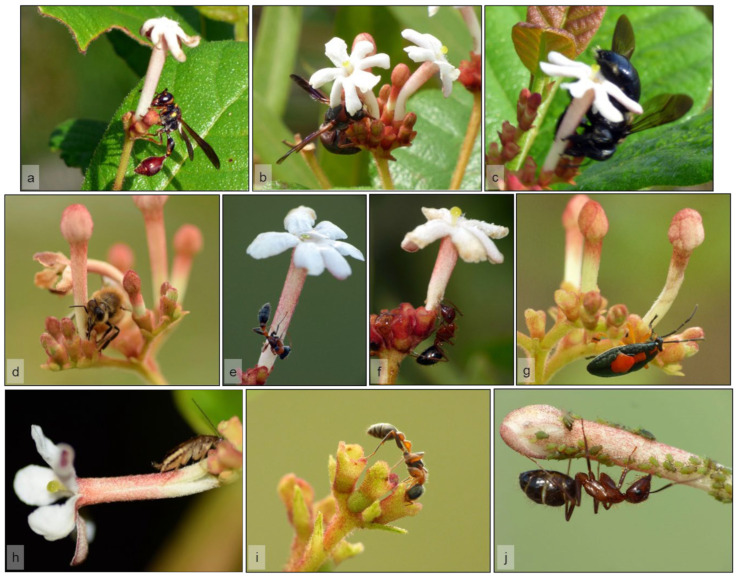
Overview of nectar robbers found on flowers of *Guettarda scabra* in pine rockland habitats in south Florida, USA. Diurnal primary nectar robbers such as the wasps (**a**) *Zethus slossonae*, (**b**) *Stenodynerus* sp., and the bee (**c**) *Xylocopa micans* use their mandibles to pierce the base of the corolla to access the nectar. Notice how the hairy underside of the abdomen in *X*. *micans* comes in close contact with the stigma of the flower, as the bee cuts the corolla tissue, potentially leading to pollen transfer. Secondary nectar robbers such as the honeybee (**d**) *Apis mellifera*, the ants (**e**) *Pseudomyrmex gracilis* and (**f**) *Camponotus floridanus*, the true bug (**g**) *Largus succinctus* (nymph), and (**h**) a cockroach (*Blattodea* sp.), drink nectar through holes cut at the base of the corolla tube by a previous visitor. Opportunistic ant visitors such as *P*. *gracilis* can also feed on nectar from postfloral nectaries (**i**) or as observed in *C. floridanus*, feed on honeydew secreted by aphids (**j**).

**Figure 6:**
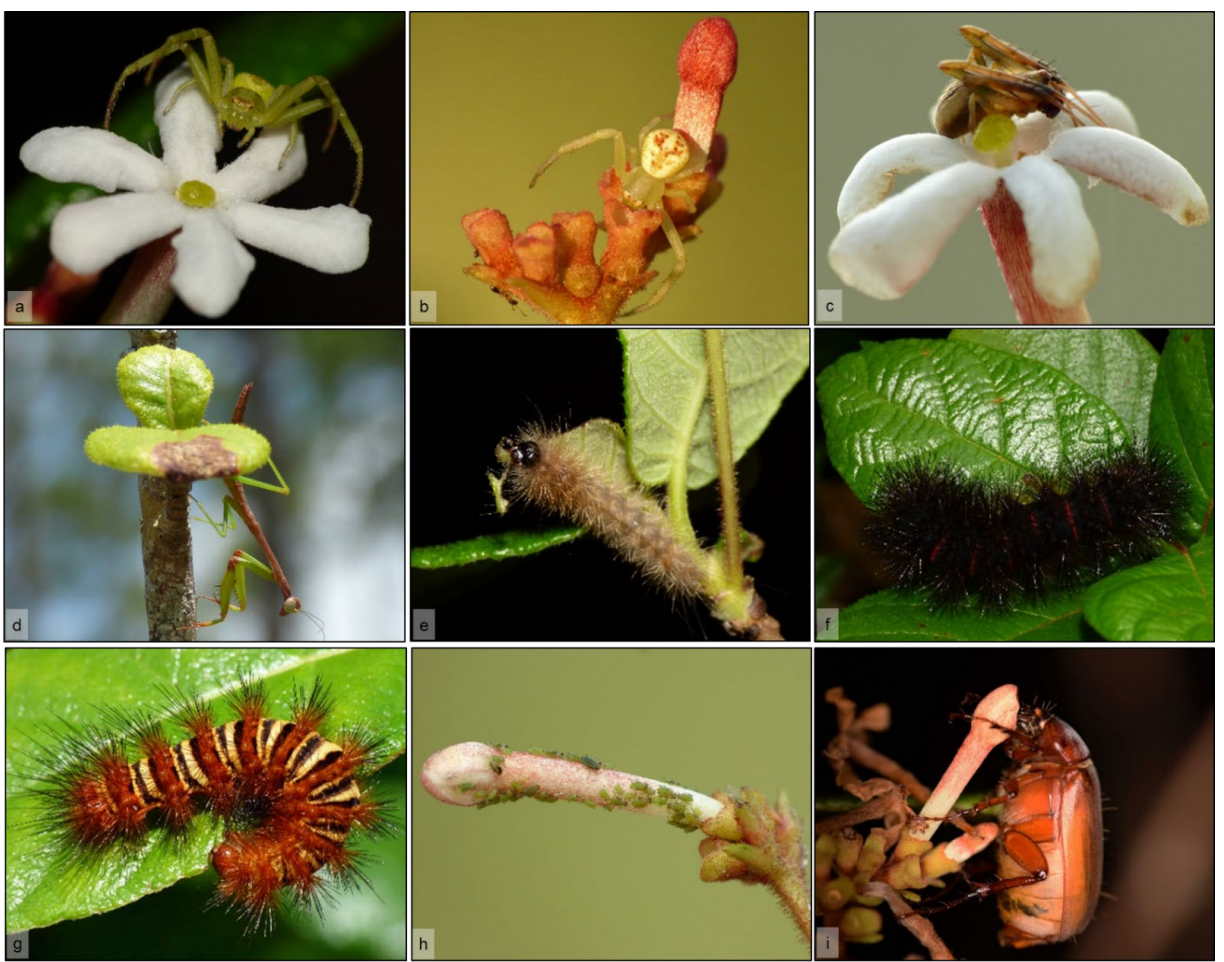
Overview of predatory and herbivorous arthropods on *Guettarda scabra* in pine rockland habitats in south Florida, USA. (**a**) Crab spiders of the genus *Mecaphesa* in hunting position on a corolla, and (**b**) on an unopened bud. (**c**) Orbweaver spider *Acacesia hamata* sitting on an open flower almost touching the exposed stigma. (**d**) Praying mantis *Stagmomantis floridensis* exploring a branch in the morning. Caterpillars of the erebid moths (**e**) *Calidota laqueata*, (**f**) *Hypercompe scribonia* and (**g**) *Seirarctia echo*, found consuming leaves of *G. scabra*. Other herbivores found associated with flowers include (**h**) clusters of aphids sucking sap from a flower bud, and a (**i**) May beetle *Phyllophaga* sp. chewing on a flower bud at night.

We identified four behaviors among arthropods visiting *G. scabra* flowers: (a) legitimate visitation, consumption of pollen or nectar through the opening of the corolla tube involving contact with the anthers, stigma, or both and potentially resulting in pollination; (b) nectar robbing, consumption of nectar through a perforation of the corolla either made by the visitor itself (primary robber) or left by a previous visitor (secondary robber); (c) predation on other arthropods; and (d) herbivory, feeding on leaves or flowers (Table 1, Figure 4, Figure 5 and Figure 6). 

**Figure 7:**
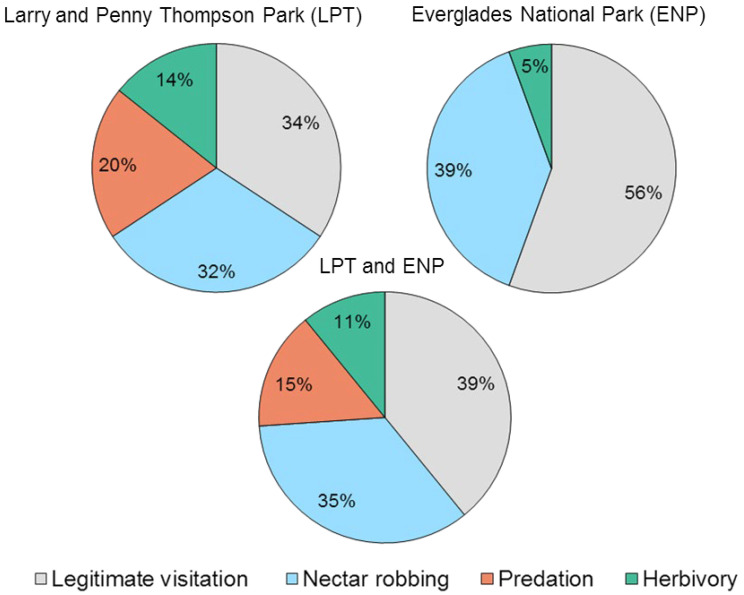
Relative occurrence of the four visitor behaviors observed on flowers of *Guettarda scabra*, among arthropod species in pine rockland habitats in south Florida, USA. Percentages represent the fraction of species observed engaging in a particular behavior on flowers, with respect to the total number of species found in Larry and Penny Thompson LPT (35 species), in Everglades National Park ENP (18 species), or in both sites combined (46 species).

Overall, legitimate visitation and nectar robbing were the most common behaviors observed among the flower-visiting species (Figure 7). Fisher’s exact test showed those behaviors combined were substantially greater than the others combined (*p* < 0.05), but neither was significantly different from the other, nor were predation and herbivory different from one another. The same patterns were seen at both sites separately. More than half (56%) of flower visitors at ENP and 34% at LPT visited flowers legitimately, and these were mainly lepidopterans (Table 1, Figure 4). Nectar robbing was performed by 32% of the visitors observed at LPT, and by 39% at ENP, mostly Hymenoptera (Table 1, Figure 5). Of the nectar robbers, 75% acted as secondary nectar robbers (Table 1). Herbivory was performed by different groups of insects at both study sites (Table 1, Figure 6), while predation was only witnessed at LPT and performed by spiders and mantises (Table 1, Figure 6).

### 2.3. Resources Consumed by Visitors

**Figure 8:**
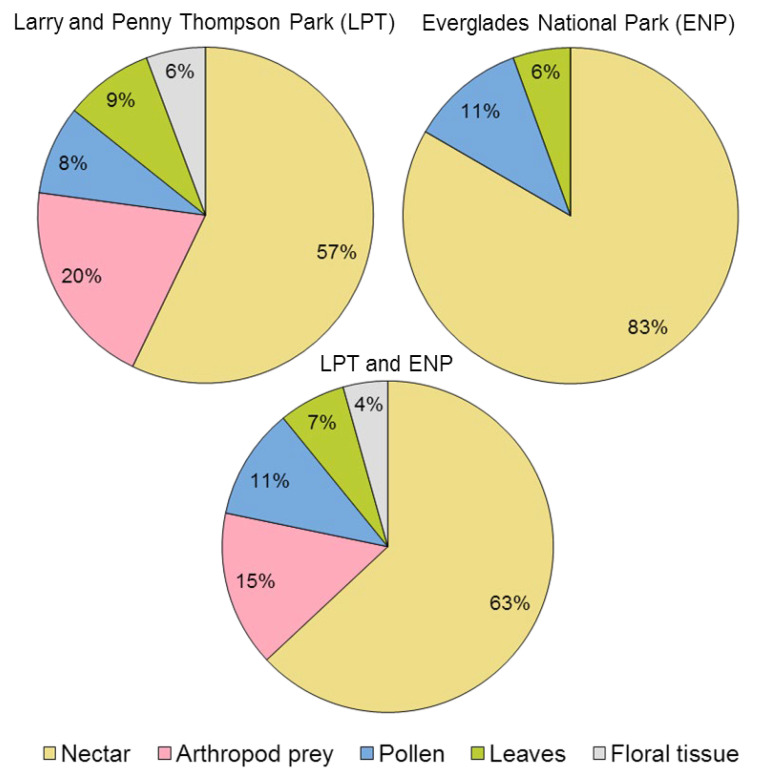
Relative usage of plant resources provided by *Guettarda scabra*, among arthropods in pine rockland habitats in south Florida USA. Percentages represent the fraction of species benefiting from a particular resource, with respect to the total number of species found in Larry and Penny Thompson LPT (35 species), in Everglades National Park ENP (18 species), or in both sites combined (46 species).

Visitors obtained five types of resources from *G. scabra* plants: nectar, pollen, floral tissue, leaves, and small insects attracted to the plant serving as prey (Table 1). Nectar was by far the main resource consumed by the arthropod community overall (Fisher’s exact test, *p* < 0.01) as well as in both ENP (*p* < 0.01) and LPT (*p* < 0.01) (Figure 8), mostly Lepidoptera and Hymenoptera (Table 1). A surprising result was that some insects consumed post-floral nectar secreted after the corollas fell, the first time this has been observed in *G. scabra*. Consumption of other resources involved 43% of visitor species at LPT and only 17% of them at ENP (Figure 8). Just as with predation, we did not witness any visitors feeding on floral tissue at ENP.

### 2.4. Taxonomic Diversity and Behaviors of Visitors

#### 2.4.1. Lepidoptera

This order contains 15 of the 46 species found visiting flowers of *G. scabra* in both study sites, making it one of two orders of arthropods with the greatest species richness attracted to these flowers. Among Lepidoptera, 11 were skippers (Hesperiidae), butterflies (Nymphalidae, Papilionidae), and moths (Erebidae), while the remaining were hawkmoths (Sphingidae) (Table 1). Among the 15 species of Lepidoptera found, 5 were observed in both study sites (e.g., Figure 4a–c), 7 were seen only in LPT (e.g., Figure 4d–e), and 3 only in ENP (Figure 4f–h) (Table 1).

While most Lepidoptera visited flowers during the day, the hawkmoths (*Eumorpha fasciatus, Perigonia lusca*, and *Xylophanes tersa*) were observed exclusively at night (Figure 3). In general, hawkmoths approached the plants by flying fast through the vegetation and fed only on fresh flowers by hovering above the corolla with their proboscis extended. Moths tended to visit a couple of flowers per plant and then fly away, maintaining a low number of visits per night. Individuals of *E. fasciatus* were often seen hanging motionless on branches of different plants around 2130 h, after visiting flowers (Figure 4h).

In contrast to hawkmoths, butterflies and skippers were observed foraging more frequently and visiting most of the flowers available in a single plant before moving to a nearby individual. Their intensive foraging strategy often resulted in multiple individuals and species feeding simultaneously on a single plant, occasionally even on withered flowers. Butterflies and skippers fed by landing on flowers and inserting their proboscis, and at times part of their head, into the corolla tube to reach the nectar (Figure 4a–f), sometimes resulting in large amounts of pollen being deposited on their mouthparts.

Besides adult lepidopterans, caterpillars of the erebid moths *Calidota laqueata*, *Hypercompe scribonia*, and *Seirarctia echo* were found feeding on leaves of *G. scabra* (Figure 6e–g). None of the adults of these species were observed visiting flowers. 

#### 2.4.2. Hymenoptera

Hymenoptera was the other order with many species visiting flowers of *G. scabra* (30.4% of all recorded visitors). Over half of them (57%) were found exclusively at LPT and only one species (*Zethus slossonae*) observed in both study sites (Table 1). Most Hymenoptera observed were either wasps or bees, while ants were represented by only a few species (Table 1). The ants *Pseudomyrmex gracilis* (Figure 5e) and *Camponotus floridanus* (Figure 5f) were the most frequently found throughout this study (Figure 3), although only at LPT. Notably, both the carpenter bee *Xylocopa micans* (Figure 5c) and the honeybee *Apis mellifera* (Figure 5d) were a common sight in ENP, in contrast to the remaining species.

Hymenopterans were observed to be active exclusively during the day, except for *C. floridanus* which foraged during the night as well (Figure 3). All hymenopterans visiting flowers of *G. scabra* fed on nectar, except for *Dielis trifasciata* (Figure 4i, Table 1) that consumed only pollen by inserting its head into the corolla opening. *Euglossa dilemma* was the only hymenopteran feeding on nectar through the natural opening of the flower while hovering over it, whereas *X. micans*, *Z. slossonae*, and an unidentified vespid wasp (*Vespidae* sp. 1) actively pierced the base of the corolla to access nectar (Figure 5a,c). Notably, individuals of *X. micans* observed during this study landed on the flowers and positioned themselves facing the base of the corolla, with their abdomen directed toward the flower opening. As the large bee cut the corolla tube, its abdominal hairs were rubbed against the anthers and sometimes the stigma (Figure 5c).

Remaining Hymenoptera acted as secondary nectar robbers, except for the wasp *Stenodynerus* sp. (Figure 5b), the only species behaving as both primary and secondary nectar thief. Ants moved through the plants constantly, exploring flowers to feed on nectar even after corolla abscission, upon which they visited post-floral nectaries (Figure 5i). Whenever scale insects or aphids were present, ants were seen protecting them and feeding on honeydew, which led to some aggressive interactions observed in LPT between *Wasmannia auropunctata* and *P. gracilis*.

#### 2.4.3. Coleoptera

Flowers of *G. scabra* at ENP were visited at night by two species of long-horned beetles (Cerambycidae) that fed on pollen: *Eburia stigma* (Figure 4j) and *Plectromerus dentipes*. These beetles flew through the vegetation visiting one or two flowers per plant, where they were seen feeding on secretions of the stigma and inserting their head into the corolla to reach the pollen on the anthers (Figure 4j). In contrast, plants at LPT were visited by two species of scarab beetles (Scarabaeidae) (Table 1). *Euphoria sepulcralis* (Figure 4k) fed on pollen during the morning (Figure 4k), while *Phyllophaga* sp. was observed consuming open flowers and large buds at night, after 2100 h (Figure 6i).

#### 2.4.4. Other Insect Orders

Observations of flies visiting flowers of *G. scabra* were uncommon (Table 1). There was a single morning sighting of the flower fly *Ornidia obesa* at LPT, during which the fly hovered before landing on flowers to consume pollen through the natural opening of the corolla, contacting the exposed stigma with its mouthparts in the process. Additionally, two species of crane flies (Tipulidae) were found acting as secondary nectar robbers during the day, feeding through holes available at the base of the corolla at both study sites.

The only Hemiptera observed at both study sites were *Largus succinctus*, a secondary nectar robber (Figure 5g). Occasional observations of aphids (Aphididae) extracting sap from flowers and buds only occurred at LPT (Figure 6h). Aphids were often accompanied by *C. floridanus* ants (Figure 5j), and in one instance also by a silver fly *Leucopis* sp. (see [16]). Scale insects (Coccoidea) were also found on inflorescences at LPT, but their presence was not recorded systematically.

Finally, two groups of Orthoptera were found only at LPT: a species of cockroach (Blattodea) acting as a secondary nectar robber and also visiting post-floral nectaries at night (Figure 5h); and two species of praying mantises (Mantidae) perched at the base of the inflorescences during daytime (Figure 6d).

#### 2.4.5. Aranae

Five species of spiders were observed during this study, all of them at LPT: the orbweaver spider *Acacesia hamata* and four crab spiders of the genus *Mecaphesa* (Table 1). All spiders were observed sitting on the corolla, close to the pistil in both fresh and withered flowers, as well as on inflorescences with unopened buds (Figure 6a–c). They were observed either capturing small insects or resting on a flower with their front legs held out to each side of their body, a characteristic pose in this group.

## 3. Discussion

Although it was previously assumed that *G. scabra* is a moth-pollinated plant, our findings show that their flowers are visited by a wide array of arthropods that can act as pollinators, most of them diurnal. Such diversity is not surprising, since nearly 30% of arthropods species visit flowers regularly and potentially pollinate them [17]. Likewise, differences in diversity of visitors between night and day occur in many other plants whose flowers exhibit sphingophily, particularly the diurnal dominance of Hymenoptera and Lepidoptera that we observed in *G. scabra* (e.g., [18,19,20,21]), both groups being the largest insect taxa containing important pollinators [17]. 

In general, night-blooming species whose flowers remain open into the morning may be attractive to diurnal visitors, especially those unvisited flowers that accumulated nectar through the night [22]. Diurnal visitation of nocturnal flowers by a variety of animals has been reported across different families of plants. Examples highlighting the taxonomic diversity of plants include species of the families Caprifoliaceae and Cactaceae that are visited by bees [19,23], Apocynaceae and Rubiaceae by bees and butterflies [18,24], and Bromeliaceae by bees, ants, and flies [25]. The availability of nectar in the morning can even attract hummingbirds, as observed in Bromeliaceae [25] and Rubiaceae [26]. In the latter family, the genus *Guettarda* contains several species with this pattern of anthesis in which sphingophylous flowers remain open through the morning making nectar and pollen available to diurnal visitors. Observations on *G. speciosa* in south China [27] and *G. clarensis* in Cuba [28] revealed that both species were visited by a single local species of nocturnal hawkmoth and some diurnal insects, mostly lepidopterans, hymenopterans, and dipterans. While these two species were visited by both nocturnal and diurnal insects, *G. platypoda* in Brazil was solely visited by nocturnal moths of three species [29]. These observations contrast with our findings, since *G. scabra* flowers are visited by a much larger number of species during the day and night. However, the frequency of visits by nocturnal hawkmoths was as low as in *G. platypoda* [29] and *G. speciosa* [27] (M.C.P. unpublished observations). Attracting a larger set of flower visitors may be advantageous for *G. scabra*, as non-hawkmoth visitors may provide some pollination when specialized hawkmoth pollinators are not available. 

### 3.1. Occurrence

Our findings suggest that the flowers of *G. scabra* are visited by a community of arthropods whose structure differs between study sites. These differences may be linked to variations in the availability of biotic components of the ecosystem that depend on the presence of particular species of arthropods. Some of the species visiting flowers of *G. scabra* may require other resources that can vary between study sites, such as the presence of host plant species in the case of Lepidoptera, or nesting and shelter spaces for other arthropods. Carpenter bees (*X. micans*), for example, rely on the availability of dead wood they need to build their nests [30]. The scarcity of this resource might explain the absence of this species in LPT. On the other hand, our observations of the skipper *E. brunnea* in both study sites are clearly related to the availability of its host plant *Byrsonima lucida* (Malpighiaceae) [31] in both areas.

Surprisingly, the lepidopterans *Heliconius charithonia, Polites baracoa, Cymaenes tripunctus,* and *Papilio polyxenes*, which were all reported present all year round in the Long Pine Key area of ENP more than 40 years ago [31], were not observed in that area during this study, although we did observe them visiting flowers in LPT. Other notable absences in ENP include the caterpillars of three erebid moths (*Seirarctia echo*, *Spilosoma virginica,* and *Pyrrharctia isabella*), a paper wasp (*Mischocyttarus* sp.) and a species of flower fly (*Copestylum mexicanum*) seen visiting flowers of *G. scabra* over 30 years ago [14]. However, *S. echo* and a species of *Mischocyttarus* were found on *G. scabra* in LPT.

Interestingly, almost 25% of the total number of arthropod species found visiting flowers of *G. scabra* were only at ENP, a site that we undersampled with respect to LPT. While the sampling effort was different enough between both sites to prevent us from drawing any solid conclusions, the high proportion of species found only in ENP suggests that the communities of floral visitors are indeed different between study sites. It is possible that the arthropod community associated with *G. scabra* flowers in south Florida is even more taxonomically diverse than reported here. 

### 3.2. Potential Pollinators

*Guettarda scabra* is visited by a wide range of potential pollinators besides lepidopterans. In fact, plants whose flowers fit a particular pollination syndrome may still receive visits from opportunistic insects capable of contributing to their fitness [3,23,32].

Due to floral morphology in *G. scabra*, most of their visitors with short mouthparts (such as bees, wasps, flies, and beetles) encounter anthers, stigma, or both while foraging, potentially serving as pollen vectors for this plant. Since anthers in flowers (of all morphs) of *G. scabra* are located at the opening of the corolla, short-tongued visitors can access pollen grains in any open flower and may then transfer them to flowers with exserted stigmas. Successful pollination of flowers with long corollas by short-tonged insects has been observed in other distylous Rubiaceae, such as *Psychotria homalosperma*. While that plant is presumably pollinated by long-tongued moths, in their absence, honeybees (*Apis mellifera*) manage to pollinate it with their short mouthparts by moving pollen unidirectionally from short- to long-styled flowers [33]. We think that a similar scenario occurs in *G. scabra*, where both short- and long-tongued visitors may promote pollination.

While short-tongued visitors could contribute to reproduction in *G. scabra*, floral traits in this species suggest the existence of a most effective pollinator with long mouthparts capable of encountering the stigma, regardless of how deep in the corolla it is located. Regarding the identity of such pollinator, previous authors have found nocturnal hawkmoths to be the main pollen vectors for other species in the genus *Guettarda*, such as *G. platypoda* [24,29], *G. speciosa* [27], and *G. clarensis* [28]. In fact, the nocturnal hawkmoth *X. tersa* was a common flower visitor of *G. scabra* during this study, and the same hawkmoth was the most frequent pollinator for *G. platypoda* in Brazil [29], suggesting a particular association between this moth and *Guettarda* plants when both are present. Besides lepidopterans, the bee *E. dilemma* was the only other visitor with a tongue long enough to reach nectar deep in the corolla of the flowers of *G. scabra*. This bee has a mutualistic relationship with orchids in its Central American native range, and was recently introduced to south Florida, where it has been reported (as *E*. *viridissima*) visiting a wide variety of non-orchid plants [34]; (Brittany M. Harris, personal communication). Our study provides the first record of *E. dilemma* visiting flowers of *G*. *scabra*.

Attracting different types of potential pollinators could enhance fruit production in areas where the most effective pollinator is absent or scarce [19], and/or when weather conditions disrupt foraging activity [35]. This plant has survived in the highly fragmented pine rockland habitat, being regularly exposed to extreme weather events such as heavy rainfall and flooding, hurricanes, and fire. Despite the low frequency of visits by nocturnal hawkmoths locally (M.C.P. unpublished observations), flowers of *G. scabra* may increase their chances of being pollinated by receiving visits from other pollen vectors observed during this study. In fact, day-active flower visitors may complement the effect of nocturnal ones in this species, as has been suggested by indirect observations [36].

### 3.3. Nectar Robbing: A Common Behavior

The fact that nectar robbing was a very common behavior observed among the floral visitors of *G. scabra* agrees with other instances in which more than half of the species of flower visitors are nectar robbers [37]. This behavior is known to happen in other species of the genus *Guettarda*. In *G. clarensis,* for example, nectar robbing reduces fruit production, negatively impacting reproduction [38]. Interestingly, the main robbers in *G. clarensis* (*Largus sellatus* and *Xylocopa cubaecola*) belong to the same genera as two common robbers we found in *G. scabra* (*L. succinctus* and *X. micans*) [39]. Nectar robbing has also been reported in *G. speciosa* [27], but there is no detailed account of these observations. In *G. scabra*, we did not observe damage caused by nectar robbers on sexual structures of the flowers (i.e., pistil or stamens), which could directly interfere with pollination, but it is unknown whether robbing can affect reproduction in this species.

While nectar robbing may be detrimental for plant reproduction [40], under certain conditions it may also have positive effects [41], such as contributing to pollination. Some *Xylocopa* bees for example, have been reported robbing nectar from plants with long tubular flowers [33,42,43,44,45,46]. In certain cases, they have been seen touching the anthers and stigma of flowers as they feed, promoting pollination [44,45,46,47]. Our observations on the foraging behavior of *X. micans* suggest that these bees may transfer pollen in *G. scabra* during nectar robbing. However, no other nectar robber observed during this study behaved or positioned its body in a way that could result in pollen transfer while they were feeding.

In addition to robbers depositing pollen, they may benefit the plants they rob in another way: by causing floral visitors to visit fewer flowers and move to other plants more quickly [48]. This is especially beneficial in plants that are self-incompatible [49] but may be important in avoiding inbreeding depression in those that are self-compatible as well by reducing geitonogamy [50]. As *G. scabra* is self-compatible, it may benefit from the actions of its numerous nectar robbers. 

### 3.4. Guettarda scabra as Food Source for Local Arthropod Fauna

Floral resources can be a limiting factor in many habitats during a particular season. In pine rocklands, most species flower from January to April [51] and initiate fruit formation during summer [52], reducing the availability of floral resources during this time. In contrast, most *G. scabra* individuals are fully in flower in June and July, when few other species are flowering, making them a valuable source of floral rewards. Our findings suggest that *G. scabra* may be a keystone species in the pine rockland habitat of south Florida, as it is an important source of food and foraging grounds for the local arthropod fauna during its flowering season. Flowers of this plant provide highly nutritious resources in the form of pollen and nectar to visitors, as well as flower parts and leaves for herbivores, making this plant attractive to a large variety of arthropods with diverse natural histories. 

In fact, *G. scabra* flower rewards are used by wasps found only in Florida, such as *Z. slossonae* [53] and *D. trifasciata* [54]. Flowers of *G. scabra* also provide nectar for adult lepidopterans with distributions restricted to the southern half of Florida, such as *Perigonia lusca* [55] and *Cymaenes tripunctus* [56], along with *Ephyriades brunnea* whose populations have declined in recent years [57]. Such a critical role in the maintenance of the local pollinator fauna was observed also in *G. platypoda* in Brazil, where hawkmoth communities rely on its nectar as an energy source [24]. Although most adult lepidopterans visit *G. scabra* to feed on nectar, *H. charithonia* probably also consumes pollen, a resource reported as part of its diet [58,59,60]. Besides the erebid moths reported in this study, *G. scabra* is the host plant for caterpillars of other species of moths in south Florida, such as *Spilosoma virginica* and *Pyrrharctia isabella* [14], as well as the hawkmoths *P*. *lusca* and *Eupyrrhoglossum sagra* [55,61]. 

Our observations also suggest that the pollen of *G. scabra* is an important food source for local populations of some long-horned and scarabeid beetles. In fact, scarabeids may rely on more than pollen from this plant, since at least *Phyllophaga* sp. was observed consuming its flowers during this study. It is also possible that *Euphoria sepulcralis* feeds on flower tissue of *G. scabra* as well, based on field observations of this species consuming flowers of other plants in LPT, including *Bidens* sp. (Asteraceae), *Spermacoce* sp. (Rubiaceae), and *Lantana* sp. (Verbenaceae) (M.C.P. personal observations), and occasional reports of this species as flower-damaging pest in some fruit trees in south Florida [62].

Besides insects, spiders may spend time on flowers benefiting from food sources other than prey. Spiders can feed on stigma exudates, nectar, and pollen [63,64,65,66,67,68]. While we did not witness this behavior directly, we often saw individual spiders sitting on the corolla, with their mouthparts very close to the stigma, anthers, or postfloral nectaries. Considering that the stigma of *G*. *scabra* remains moist throughout anthesis, and even after the corolla tube is wilted, spiders may have been feeding on stigmatic exudates. Interestingly, most of the spiders observed on flowers of *G. scabra* belong to the family Thomisidae, a group also commonly observed on flowers of *G. clarensis* [28].

The effect of predatory visitors on the reproductive success of *G. scabra* is unknown. In general, predators can harmfully disrupt pollination by consuming pollen vectors [69,70] or decreasing the frequency and duration of their visits [71,72,73,74,75]. Sometimes predators may benefit plants by causing pollinators to move between plants more [76], promoting outcrossing [77] as can nectar robbers [48]. At the same time, they can benefit the plant by decimating insects feeding on it [78]. In fact, some of the wasps observed during this study are known to attack phytophagous larvae, such as *Pachodynerus erynnis* that feeds on caterpillars of several families [79,80], or *D*. *trifasciata* which parasitizes larvae of the beetle *Phyllophaga portoricensis* [81]. Interestingly, we found a species of *Phyllophaga* consuming flowers of *G. scabra*, raising the question of whether *D. trifasciata* can control the local population of this beetle and benefit *G. scabra* in the process.

## 4. Materials and Methods

### 4.1. Plant Species

The rough-leaf velvetseed *Guettarda scabra* (Rubiaceae) is a tropical shrub usually less than 1.5 m tall when it grows in pine rockland forests in south Florida. Its blooming season begins in April and peaks between May and July [12]. Plants resprout after fire, but do not bloom the summer after burning, taking two years from fire until blooming again [36]. Flowers are white, often with a pink-flushed corolla tube, about 2 cm long that holds nectar at its base (Figure 1a). Flowers are arranged in dichasial cymose inflorescences and open sequentially over several weeks, usually one to three flowers per inflorescence per day, releasing a strong, sweet scent. Anthesis occurs during late afternoon and flowers remain fresh through the following morning [10]. Flower senescence occurs usually by noon, when the corolla turns brown and dehydrates, remaining attached to the calyx for about a day [10]. 

*Guettarda scabra* exhibits a special case of distyly, in which both the anther height and style length vary continuously in the population [10]. Plants are self-compatible, sometimes setting fruit without visitation, but pollen vectors are required for greater fruit production [10]. 

### 4.2. Study Sites

This study was conducted in two natural areas in Miami-Dade County, Florida, USA: (a) Larry and Penny Thompson Memorial Park (LPT), a county park containing the largest fragment of pine rockland habitat in the city of Miami (25°35′55″ N 80°23′55″ W); and (b) the Long Pine Key area (25°24′13.2″ N 80°39′33.2″ W), within a large, continuous pine rockland forest in Everglades National Park (ENP) (Figure 1b). The pine rockland habitat is unique to south Florida and the Caribbean and is considered critically imperiled due to a substantial loss of its original extent [82,83]. Although the objective of this study was not to compare the two sampling sites, for some aspects the data are shown separately to discuss general trends.

Rockland habitats are greatly reduced from their original extent as they have undergone extensive human development over the last century [83,84,85]. Pine rocklands are considered globally imperiled [86] with many endemic plant taxa in the diverse understory of more than 225 native plant species, of which 10% are considered threatened or endangered at the state level, eight of which are federally endangered [87].

### 4.3. Flower Visitor Observations

We surveyed arthropods visiting *G. scabra* flowers and/or feeding on the plant during the blooming seasons of 2016, 2018, and 2019 (17, 3, and 31 days respectively) at LPT, and during 2018 and 2019 (5 and 3 days respectively) at ENP. Observations were carried out on groups of plants with open flowers for 30 min at a time, three times a day. Surveys done between 0700–1200 h were considered to have been performed in the morning, 1800–2019 h in the evening, and 2020–2300 h at night. Nocturnal observations were made using red light lanterns to minimize disturbing the behavior of insect visitors. A total of 75 of these observation periods were conducted in LPT (48 mornings, 20 evenings and 7 nights) on 25 plants, and 11 in ENP (2 mornings, 5 evenings and 4 nights) on 20 plants. Additionally, visitors spotted on flowers of *G. scabra* incidentally while walking through the study sites were recorded. The data reported are the number of observation periods in which each type of visitor was observed. 

All arthropods observed touching flowers were considered floral visitors. Due to the potential relevance of lepidopterans in the pollination biology of *G. scabra*, caterpillars feeding on plants were documented, collected, and reared for species determination. Flower visitors were recorded, noting their time of activity and behavior (harvesting reward, contacting sexual organs of the flower, and interacting with other species), and photographed if possible. When necessary, voucher specimens were preserved to confirm identification. These specimens will be deposited in the Florida State Collection of Arthropods (Gainesville, FL, USA).

### 4.4. Statistical Comparisons

To evaluate the relative importance of different groups of visitors, their behaviors, resources utilized, and activity periods, we used Fisher’s exact test (which is appropriate for small sample sizes) to compare the numbers of species associated with each of those parameters. We used a significance level of *p* < 0.05 for single comparisons and *p* < 0.01 for multiple.

## 5. Conclusions

Although *G. scabra* flowers have traits traditionally associated with attracting nocturnal moths, they open in the evening and remain open into the morning, luring in a much wider array of floral visitors. Despite recent work on the diversity of flower-visiting arthropods in the Everglades [88,89,90] and pollination of plants in the pine rockland habitat [91,92,93,94,95,96], little is known about the entire array of flower visitors to any particular plant species. The maintenance of healthy pine rockland habitat requires periodic fires to prevent succession to hardwood hammock forest [84], and in the open pine rockland understory *G. scabra* grow relatively free of competition from other hardwoods, investing much energy into flowering [6]. This study constitutes the first in-depth survey of insects and arachnids associated with the abundant flowers of *G. scabra* in this habitat. 

Our findings show that *G. scabra* is not only visited by nocturnal hawkmoths as expected, but many other potential pollen vectors, beyond those predicted by its pollination syndrome. Our observations also suggest that this plant provides an important foraging and food resource for the local arthropod fauna. Our research provides baseline data on the local arthropod fauna associated with a native plant species, along with insights into the complexity of trophic interactions in the pine rockland habitat. There are 147 recognized species of the genus worldwide [97], but no species of *Guettarda* are considered rare, and those that are ranked by conservation organizations are apparently secure, the habitats in which many occur are imperiled or unranked and threatened in ways similar to the pine rocklands. The richness of floral visitors to *G. scabra* and the critical role this plant may play in sustaining that community indicates that plants may host a wide array of arthropods, regardless of the presence of adaptations suggesting coevolution with a much narrower set of visitors. Our observations on the natural history of *G. scabra* offer a glimpse of how intricate plant-animal interactions can be. For threatened habitats such as the pine rocklands in south Florida, studies like this yield needed information to support efforts to conserve and protect them along with their associated diversity of plants and animals. 

## Figures and Tables

**Table 1:** Array of arthropods associated with *Guettarda scabra*, their behaviors, and plant resources used at two pine rockland sites (Larry and Penny Thompson Memorial Park: LPT; and Long Pine Key, Everglades National Park: ENP) in south Florida. Observed behaviors abbreviated as follows: predation on other arthropods (pr), legitimate visitation (lv), primary nectar robbing (1nr), secondary nectar robbing (2nr), and herbivory (h). Plant resources used by visitor abbreviated as follows: arthropod prey (ap), nectar (n), pollen (p), floral tissue (f), and leaves (l). Asterisks signify caterpillar stage.

CLASS ORDER Family Species (Author)	Behavior on Plant	Resource Used	Study Site	
			LPT	ENP
ARACHNIDA				
ARANEAE				
Araneidae				
* Acacesia hamata* Hentz	pr	ap	x	
Thomisidae				
*Mecaphesa* sp. 1	pr	ap	x	
*Mecaphesa* sp. 2	pr	ap	x	
*Mecaphesa* sp. 3	pr	ap	x	
*Mecaphesa* sp. 4	pr	ap	x	
INSECTA				
BLATTODEA				
*Blattodea* sp.1	2nr	n	x	
COLEOPTERA				
Cerambycidae				
*Eburia stigma* Oliver	lv	p		x
*Plectromerus dentipes* Oliver	lv	p		x
Scarabaeidae				
*Euphoria sepulcralis* Fabricius	lv	p	x	
*Phyllophaga* sp.	h	f	x	
DIPTERA				
Syrphidae				
*Ornidia obesa* Fabricius	lv	p	x	
Tipulidae				
*Tipulidae* sp.1	2nr	n	x	
*Tipulidae* sp.2	2nr	n		x
HEMIPTERA				
Aphididae				
*Aphididae* sp.1	h	f	x	
Largidae				
*Largus succinctus* Linnaeus	2nr	n	x	x
HYMENOPTERA				
Apidae				
*Apis mellifera* Linnaeus	2nr	n		x
*Euglosssa dilemma* Bembé & Eltz	lv	n		x
*Xylocopa micans* Lepeletier	1nr	n		x
Crabronidae				
*Cerceris rufopicta* Smith	2nr	n	x	
Formicidae				
*Camponotus floridanus* Buckley	2nr	n	x	
*Pseudomyrmex gracilis* Fabricius	2nr	n	x	
*Wasmannia auropunctata* Roger	2nr	n	x	
Halictidae				
*Augochloropsis* sp.	2nr	n		x
Scoliidae				
*Dielis trifasciata* Fabricius	lv	p	x	
Vespidae				
*Mischocyttarus mexicanus cubicola* Richards	2nr	n	x	
*Pachodynerus erynnis* Lepeletier	2nr	n	x	
*Stenodynerus* sp.	1nr, 2nr	n	x	
*Vespidae* sp.1	1nr	n		x
*Zethus slossonae* Fox	1nr	n	x	x
LEPIDOPTERA				
Erebidae				
*Calidota laqueata* Edwards *	h	l	x	x
*Hypercompe scriboni* Stoll *	h	l	x	
*Seirarctia echo* Smith *	h	l	x	
Hesperiidae				
*Asbolis capucinus* Lucas	lv	n	x	x
*Cymaenes tripunctus* Herrich-Schäffer	lv	n	x	
*Ephyriades brunnea* Herrich-Schäffer	lv	n	x	x
*Polites baracoa* Lucas	lv	n	x	
Nymphalidae				
*Agraulis vanillae* Linnaeus	lv	n	x	x
*Heliconius charithonia* Linnaeus	lv	n	x	
Papilionidae				
*Papilio palamedes* Drury	lv	n		x
*Papilio polyxenes* Fabricius	lv	n	x	
Sphingidae				
*Aellopos tantalus* Linnaeus	lv	n	x	
*Eumorpha fasciatus* Sulzer	lv	n		x
*Perigonia lusca* Fabricius	lv	n		x
*Xylophanes tersa* Linnaeus	lv	n	x	x
MANTODEA				
Mantidae				
*Mantidae* sp.1	pr	ap	x	
*Stagmomantis floridensis* Davis	pr	ap	x	

## Data Availability

The data that support the findings of this study are openly available in the FIU Research Data Portal at https://doi.org/10.34703/gzx1-9v95/3BRPS3 (accessed on 19 October 2022).
